# TRPV4-Mast Cell Interactions in Neurogenic Inflammation and Chronic Diseases: A Narrative Review

**DOI:** 10.3390/ijms27062865

**Published:** 2026-03-21

**Authors:** Malak Fouani, Srishti Kumari, Anne Charles, Christopher Wickware, Ashley A. Moore, Calvin H. Cho, Soman N. Abraham, Carlene D. Moore

**Affiliations:** 1 Department of Neurology, Duke University, Durham, NC 27710, USA; malak.fouani@duke.edu (M.F.); christopher.wickware@duke.edu (C.W.); ashley.a.moore@duke.edu (A.A.M.);; 2 Department of Pathology, Duke University Medical Center, Durham, NC 27710, USA; soman.abraham@duke.edu; 3 Department of Immunology, Duke University Medical Center, Durham, NC 27710, USA

**Keywords:** neurogenic inflammation, transient receptor potential vanilloid 4 (TRPV4), mast cells, chronic diseases

## Abstract

Transient receptor potential vanilloid 4 (TRPV4) is a polymodal cation channel that is widely expressed in sensory neurons, immune cells, and structural tissues, where it integrates mechanical, osmotic, and chemical stimuli to regulate both physiological responses and disease-associated signaling. Mast cells (MCs), key immune effector cells capable of rapid mediator release through degranulation, also express TRPV4. Increasing evidence supports TRPV4-MC signaling as an important neuroimmune interface, linking mechanical and inflammatory stimuli to tissue hypersensitivity and pain. In this review, we synthesize current evidence supporting a role for TRPV4 in MC-associated neuroimmune signaling across multiple disease contexts while distinguishing settings in which TRPV4 directly regulates MC activation from those in which MC responses arise through multicellular tissue interactions. Direct TRPV4-dependent MC activation has been described in conditions such as LL-37–driven rosacea and mechanically induced inflammation, whereas in disorders including asthma, visceral hypersensitivity, bladder pain syndromes, and osteoarthritis, TRPV4 activity in epithelial, neuronal, or stromal compartments more often influences MC function indirectly through ATP–purinergic signaling, cytokine release, and neuropeptide-mediated crosstalk. Across systems, TRPV4 emerges not as a single pathogenic switch but as part of a context-dependent signaling network whose functional consequences depend on cell type, tissue microenvironment, and disease stage. Altogether, these findings identify TRPV4 as a therapeutically actionable node within neuroimmune signaling pathways and support the development of tissue-specific and combination strategies targeting both TRPV4 activity and MC-mediated signaling in chronic inflammatory and pain disorders.

## 1. Introduction

This article is a narrative review examining evidence for functional interactions between transient receptor potential vanilloid 4 (TRPV4) signaling and mast cells (MCs) in neurogenic inflammation and chronic inflammatory and pain conditions.

### 1.1. Overview of TRPV4

Transient receptor potential (TRP) channels are a family of non-selective channels involved in detecting and transducing various stimuli, including temperature, pressure, and chemical irritants [1]. TRP channels are important mediators in the sensory processes underlying pain and inflammation [2]. Among these, TRPV4, classified under the vanilloid group due to its sequence homology and shared functional features, is known for its polymodal sensitivity and role in mechanosensation, nociception, and inflammation [3,4].

TRPV4 is encoded by the TRPV4 gene located on human chromosome 12q24 [5]. The channel assembles as a homotetramer, with each subunit containing six transmembrane-spanning domains (S1–S6) and a pore loop between S5 and S6 that forms the ion-conducting pathway [6,7]. The intracellular N-terminus is characterized by multiple ankyrin repeat domains (ARDs), which are implicated in protein–protein interactions and mechanosensitivity [8]. The C-terminus contains regulatory regions, including phosphorylation sites and calmodulin-binding motifs, that modulate channel gating and interaction with signaling proteins [9].

Although initially characterized as an osmosensor, TRPV4 is now recognized as a multimodal integrator of environmental and mechanical cues, playing key roles in regulating cellular responses to diverse stimuli [10]. Its known activators include hypotonic cell swelling, matrix stiffness, shear stress, non-noxious temperatures, physiological heat (27–34 °C), and acidic environments. It responds to both endogenous ligands, such as epoxyeicosatrienoic acids (e.g., 5′,6′-EET), and synthetic agonists like 4α-phorbol 12,13-didecanoate (4α-PDD) and GSK1016790A [5,11,12].

TRPV4 is broadly expressed across diverse tissues and cell types, including the kidney, lung, heart, brain, skin, skeletal, vasculature, and sensory ganglia such as the dorsal root and trigeminal ganglia [13,14]. It is also found in specialized sensory structures, like cochlear hair cells and vibrissal Merkel cells, as well as in cell types such as keratinocytes and epithelial cells of the kidney, lungs, and gastrointestinal tract [13]. Functionally, TRPV4 mediates vasodilation in vascular endothelial cells in response to shear stress and contributes to thermosensation, mechanosensation, and osmotic pain in sensory neurons [15,16]. Additionally, TRPV4 is expressed in immune cells such as macrophages, neutrophils, and mast cells (MCs) [17].

Pathophysiologically, TRPV4 is implicated in a range of disorders, including skeletal dysplasias, arthropathies, hereditary neuropathies, and inflammatory diseases [18]. A key mechanism involves TRPV4-mediated sensitization of primary afferent neurons and the release of pro-inflammatory mediators that amplify pain signaling and contribute to the development of chronic pain conditions [19,20,21]. Overall, TRPV4 is recognized as a critical transducer of mechanical and environmental stimuli with broad physiological and pathological relevance.

### 1.2. Overview of Mast Cells (MCs)

MCs are bone-marrow-derived immune cells found in various tissues, particularly at the interfaces between the external environment and the body, such as the skin and mucosal surfaces, as well as around blood vessels [22]. MCs contain a wide range of prestored biologically active molecules, including biogenic amines, heparin or heparan sulfate proteoglycans, neutral proteases, and neuropeptides [23]. In addition, upon stimulation, they produce and explosively release many factors in a process known as degranulation. MCs are located at sites of close contact with the external environment, such as the skin, respiratory tract, and meninges, serve as critical sentinels and bridge the external environment and the immune system [24]. These mediators initiate a cascade of immune responses, including increased vascular permeability, fluid accumulation, and recruitment of additional immune cells, that collectively amplify inflammation [19,20]. Activation of MCs via various stimuli, such as allergens, neuropeptides, or mechanical triggers, leads to intracellular calcium (Ca^2+^) influx that facilitates cytoskeletal rearrangement, fusion of secretory granules with the plasma membrane, and rapid exocytotic release of preformed mediators, thereby initiating and amplifying local neuroimmune and inflammatory responses [25].

**Figure 1:**
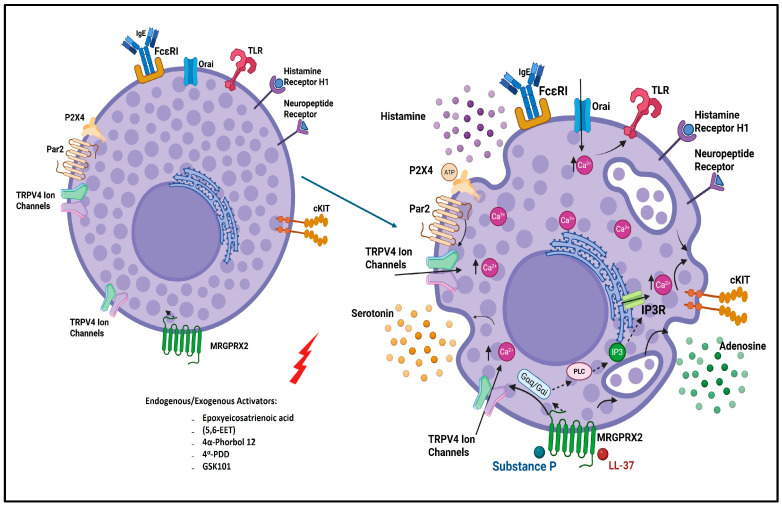
Mechanisms regulating mast cell activation and degranulation. Resting and activated MC states illustrating receptor- and ion channel-mediated mechanisms regulating MC activation and mediator release across multiple tissues and disease contexts. In the resting state, MCs express multiple surface receptors and ion channels, including FcεRI (IgE receptor), cKIT, Toll-like receptors (TLRs), histamine receptor H1, neuropeptide receptors, protease-activated receptor 2 (PAR2), purinergic P2X4 receptors, Mas-related G-protein–coupled receptor X2 (MRGPRX2), Orai channels, and transient receptor potential vanilloid 4 (TRPV4) ion channels. Activation of these receptors and channels induces intracellular Ca^2+^ influx and Ca^2+^ release from endoplasmic reticulum stores via IP_3_ receptor (IP_3_R)-dependent signaling downstream of phospholipase C (PLC). Increased intracellular Ca^2+^ promotes MC degranulation and the release of preformed and newly synthesized mediators, including histamine, serotonin, ATP, adenosine, proteases, cytokines, and antimicrobial peptides (e.g., LL-37). Neuropeptides such as substance P activate MCs via MRGPRX2, contributing to communication with sensory neurons and other resident cell types. Functional coupling between MRGPRX2 and TRPV4, PAR2 and TRPV4, and ATP-dependent signaling and TRPV4-mediated Ca^2+^ entry links neuropeptide, protease, and purinergic pathways to MC mediator release across diverse conditions. Created in BioRender accessed 5 November 2025 (https://app.biorender.com/).

Traditionally, MC degranulation has been associated with the IgE-mediated pathway, where allergens cross-link IgE antibodies bound to the high-affinity FcεRI receptor, triggering a cascade of intracellular signaling events leading to the release of inflammatory mediators. However, more recent studies have identified non-IgE-mediated pathways resulting in MC degranulation, including the activation of the Mas-related G protein–coupled receptor B2 (MrgprB2) in mice and its human ortholog MRGPRX2 [26] (Figure 1). These newly described receptors respond to a range of stimuli, including neuropeptides like substance P, and are of particular interest in neuroimmune interactions [22]. Engagement of these receptors triggers Gq-mediated phospholipase C-β (PLCβ) activation, leading to the hydrolysis of PIP_2_ into inositol 1,4,5-trisphosphate (IP_3_) and diacylglycerol (DAG) (Figure 1). IP_3_ binds to its receptors on the endoplasmic reticulum, promoting Ca^2+^ release from intracellular stores and the activation of STIM1–Orai1 store-operated Ca^2+^ entry (SOCE) channels on the plasma membrane [27,28]. Simultaneously, MrgprB2/MRGPRX2 signaling may potentiate or sensitize other Ca^2+^ channels such as TRP channels, amplifying Ca^2+^ influx from the extracellular space [29]. This combined rise in cytosolic Ca^2+^ acts as a critical second messenger that drives synaptotagmin and SNARE-dependent fusion of secretory granules with the plasma membrane, culminating in the exocytosis of histamine, proteases, and cytokines [30].

Historically, MCs are known for their role in allergic reactions, during which they release various mediators, including histamine, proteases, and cytokines, that contribute to the pathology of allergic diseases. However, MCs are now becoming recognized for their roles in angiogenesis and immune modulation during both acute and chronic inflammation and have been implicated in several neuroinflammatory disorders such as multiple sclerosis, fibromyalgia, and migraine [31,32,33]. They have an important part in neurogenic inflammation, where neuropeptides like Substance P, corticotropin-releasing hormone (CRH), and neurotensin (NT) can synergistically activate MCs to secrete pro-inflammatory compounds [34]. These mediators exacerbate local and systemic inflammation, playing a role in conditions like migraines and other neurological diseases. Studies using MC-deficient mice in a sickle cell anemia pain model have shown reduced pain sensitivity, suggesting that MCs contribute to pain pathways, likely through the release of inflammatory mediators [34,35]. Indeed, studies using MC-deficient mice in a sickle cell anemia pain model have shown reduced pain sensitivity, suggesting that MCs contribute to pain pathways, likely through the release of inflammatory mediators [35]. MCs contribute significantly to migraine pathophysiology due to their presence in the meninges, where they are closely associated with blood vessels and sensory nerve endings. This localization allows them to interact with the trigeminal system and release mediators that sensitize nociceptive neurons [36].

## 2. Mast Cells and TRPV4

MCs express multiple TRP channels, including TRPV4. Activation of TRPV4 has been shown to promote Ca^2+^ influx, associated with MC degranulation and the release of inflammatory mediators in certain experimental contexts [37,38]. Inhibition or knockdown of TRPV4 reduces MC activation in response to specific ligands, indicating that TRPV4 can regulate MC activation under defined inflammatory conditions [37,39]. TRPV4 expression is also upregulated during inflammatory states, and its activation under varying temperature conditions may contribute to MC responses in heat- or cold-induced skin inflammation [37].

However, evidence from primary MC populations indicates that TRPV4-dependent signaling is not uniform across MC subtypes or activation pathways. Studies in primary mouse peritoneal MCs demonstrate that although TRP channels, including TRPV4, are expressed, TRPV4 does not participate in FcεRI- or MRGPR-mediated activation pathways or in responses to thermal or osmotic stimulation, indicating that TRPV4 expression alone does not necessarily predict functional involvement in MC activation [40]. These findings suggest that the contribution of TRPV4 to Ca^2+^ signaling and degranulation varies across experimental models, tissues, and inflammatory contexts, and that TRPV4-dependent MC activation is likely context-dependent rather than a universal feature of MC biology.

Across the disease contexts discussed in this review, TRPV4 signaling is therefore not proposed as a uniform or MC-specific pathway but rather as a context-dependent component of broader neuroimmune signaling networks. Available evidence suggests that TRPV4 activation can influence Ca^2+^-dependent signaling within multiple cell types, including MCs, neurons, and epithelial cells, with downstream effects that may converge on inflammatory amplification, neuropeptide release, and tissue sensitization. However, the extent to which these effects reflect direct TRPV4-dependent MC activation versus indirect tissue- or neuron-mediated mechanisms varies between experimental systems and disease models. Accordingly, this review integrates findings across conditions to highlight recurring signaling themes while also identifying where MC-specific mechanisms remain uncertain or incompletely defined.

In this review, we first focus on TRPV4-dependent MC activation in rosacea, decompression sickness, and acupuncture-induced analgesia, and then expand to other contexts in which TRPV4 signaling is implicated but MC-specific mechanisms remain to be defined. Clarifying how TRPV4 shapes MC function across these settings may reveal new therapeutic strategies to modulate neuroimmune interactions in inflammatory diseases and pain.

## 3. TRPV4-Mediated Neuroimmune Crosstalk

### 3.1. TRPV4 Channels, Purinergic Signaling, and MCs in Acupuncture Induced Analgesia

#### 3.1.1. TRPV4 Channels and Mechanosensation in Acupuncture

Acupuncture, a cornerstone of traditional Eastern medicine, has been used for millennia to alleviate a wide range of ailments, particularly chronic and inflammatory pain [41]. While the clinical efficacy of acupuncture continues to be evaluated in Western medicine, recent scientific advances have begun to shed light on its molecular and cellular mechanisms.

**Figure 2:**
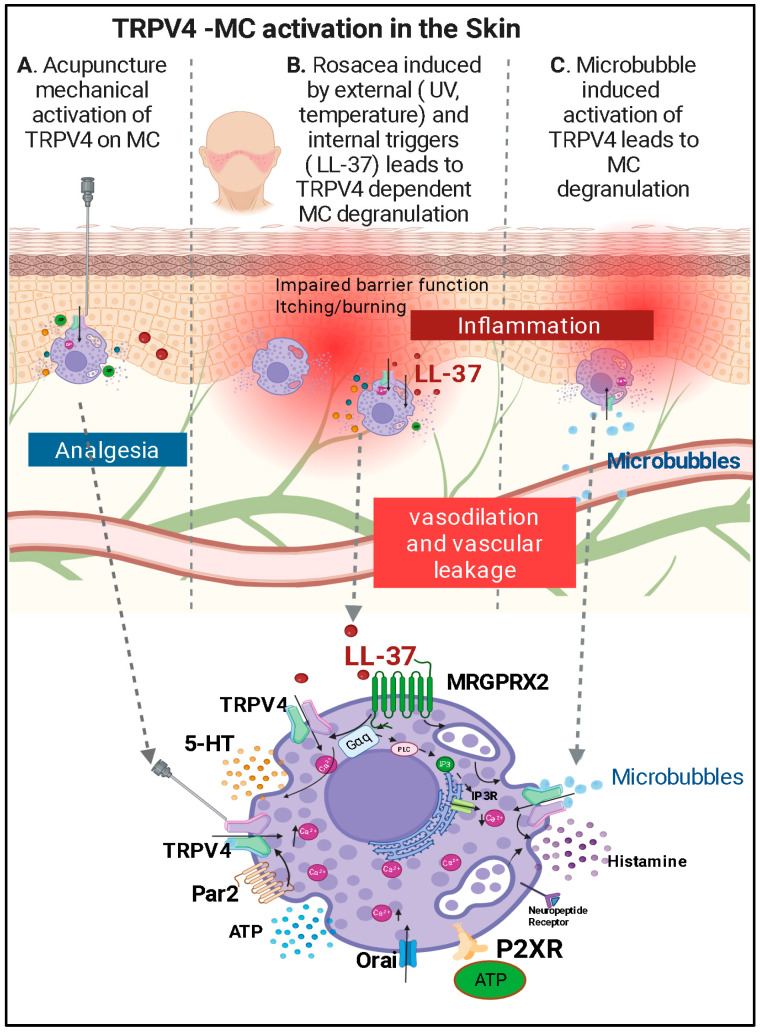
TRPV4–MC activation in the skin across distinct pathological and therapeutic contexts. (**A**) **Left** panel (Acupuncture-induced analgesia): Mechanical stimulation during acupuncture activates mechanosensitive TRPV4 channels on MCs localized near nerves and blood vessels at acupoints. TRPV4 activation promotes Ca^2+^ influx and MC degranulation, leading to the release of ATP, adenosine, histamine, and other mediators that engage purinergic signaling pathways and contribute to local analgesic effects. (**B**) **Center** panel (Rosacea): In rosacea, environmental triggers such as ultraviolet light and temperature changes, together with endogenous inflammatory mediators including the antimicrobial peptide LL-37, promote TRPV4 upregulation and activation on MCs. TRPV4-dependent MC degranulation amplifies neuroimmune inflammation, resulting in impaired skin barrier function, itching/burning sensations, vasodilation, and vascular leakage. **Right** panel (**C**) (Decompression sickness): In decompression sickness, subcutaneous microbubbles mechanically activate TRPV4 on MCs, inducing Ca^2+^ influx and degranulation. Release of histamine and other contributory mediators contributes to endothelial dysfunction, vascular leakage, and inflammatory skin manifestations. The enlarged MC inset highlights TRPV4-mediated Ca^2+^ signaling and degranulation as a shared mechanistic feature, while the tissue-level outcomes differ depending on the physiological or pathological context. (Figure created in BioRender, https://app.biorender.com/illustrations/6287f059518cc5c83015cee5, URL accessed on 20 March 2026).

Mechanical stimulation during acupuncture, especially with needle manipulation or electroacupuncture (EA), is believed to activate TRPV channels on both sensory neurons and immune cells like MCs [42]. EA has been shown to downregulate the expression and activity of TRPV channels. Lin et al. observed that EA attenuated TRPV1 and TRPV4 expression in the dorsal root ganglia (DRG) and spinal cord of a fibromyalgia mouse model, leading to reduced pain sensitivity [42,43]. This downregulation may explain the long-lasting analgesic effects of acupuncture, as sustained modulation of ion channel expression alters pain processing both peripherally and centrally (Figure 2A).

#### 3.1.2. MCs and Acupuncture

MCs are strategically located near nerves and blood vessels, especially in high density around traditional acupoints. During acupuncture, in response to mechanical stimulation, MCs become activated and undergo degranulation, releasing a cocktail of mediators such as histamine, serotonin, ATP, and adenosine [44]. Physical stimulation during acupuncture triggers ATP release from various cell types, including keratinocytes, endothelial cells, and MCs [45]. ATP is then rapidly broken down by ectonucleotidases into adenosine, which acts on A1 receptors on nociceptive neurons to further dampen neuronal excitability and sustain analgesia [44,46]. Wang et al. showed that subcutaneous MCs in acupoints trigger analgesia when activated, highlighting their role as a local amplifier of acupuncture signals [47]. Importantly, MCs express mechanosensitive ion channels, including TRPV2 and TRPV4, that facilitate this activation. This result suggests that TRPV channels are essential components of MC-mediated signaling and, by extension, the entire acupuncture analgesia pathway. Altogether, these observations support a role for MCs as local amplifiers of mechanically induced signaling during acupuncture, in which TRPV4 activation contributes to MC degranulation and mediator release that modulate local neuroimmune signaling.

#### 3.1.3. Integration of Mechanisms

The analgesic response initiated by acupuncture results from a tightly coordinated interplay between TRPV4 channel activation, purinergic signaling, and MC degranulation. Mechanical needle stimulation activates TRPV4 on MCs and sensory neurons, causing Ca^2+^ influx and ATP release. ATP acts on purinergic receptors and is rapidly degraded into adenosine, which continues to suppress nociceptive signaling via A1 receptors. Concurrently, MC mediators amplify these effects by promoting local vasodilation and increasing neurotransmitter sensitivity [46]. This process explains clinical observations such as the delayed onset and prolonged relief after a single session of acupuncture, as the release and metabolism of ATP and adenosine unfold over several minutes to hours [45]. These mechanisms provide a biologically grounded framework for acupuncture’s efficacy and offer a promising foundation for developing non-opioid pain therapies that mimic or enhance these pathways. Taken together, TRPV4 activation contributes to MC-dependent signaling during acupuncture, where TRPV4-mediated Ca^2+^ influx promotes MC degranulation and ATP release, engaging purinergic pathways that participate in modulation of nociceptive signaling.

### 3.2. TRPV4 and MC-Mediated Inflammation and Itch in Rosacea

Rosacea is a chronic inflammatory disorder characterized by episodic facial flushing, persistent erythema, visible blood vessels, and, in some subtypes, papules and pustules [48,49]. Although the exact pathophysiology remains incompletely understood, Van Zuuren et al. proposed that rosacea results from a complex interplay of dysregulated innate immunity, neurovascular dysfunction, and environmental triggers (see Figure 2B) [50,51].

Previous studies have identified MCs as pivotal contributors to cathelicidin (LL-37)–induced skin inflammation in rosacea [52]. In addition, prior work demonstrated that non-neuronal TRPV1-4 channels are differentially regulated in rosacea tissue and may mediate key disease features such as inflammation, flushing, hypersensitive skin, MC activation, and fibrosis [53]. Mascarenhas et al. investigated the role of TRPV2 and TRPV4 in regulating MC activation and mediator release in rosacea-affected skin [37]. Using primary mouse and human MCs, they found that LL-37 significantly upregulated TRPV4 expression, particularly in human MCs. Pharmacologic inhibition of TRPV4 in these MCs with the antagonist HC067047 reduced LL-37-induced MC degranulation. Further mechanistic analyses revealed that this effect was mediated through the G-protein-coupled receptor Mas-related gene X2 (MRGX2/MRGPRX2), suggesting a novel LL-37–MRGX2–TRPV4 signaling axis in rosacea. A subsequent commentary on this work by Chen et al. [54] posed two critical questions: what intracellular pathways drive the increased expression of TRPV4 in response to LL-37, and does TRPV4-mediated Ca^2+^ influx directly trigger MC degranulation?

Similarly, in an LL-37 mouse model, intradermal injections produced rosacea-like facial lesions accompanied by robust scratching behavior indicative of itch [55]. Molecular analyses demonstrated significantly increased TRPV4 expression in pruritic mouse skin, where MCs reside, as well as in corresponding trigeminal ganglia and human rosacea samples [55]. More broadly, TRPV4 has been implicated in both histaminergic and non-histaminergic itch, acting in keratinocytes, sensory neurons, and potentially MCs to drive Ca^2+^-dependent neuroimmune signaling, as comprehensively reviewed by Zhang and colleagues [56,57]. In parallel, epidermal TRPV4 activation promotes endothelin-1 (a potent MC activator and known pruritogen) signaling and cutaneous inflammation following UVB exposure, further linking cutaneous TRPV4 to pruritogenic and neurogenic pathways [14,58].

Together, these data indicate that TRPV4 participates in MC activation and degranulation in rosacea, thereby amplifying neuroimmune inflammation and itch through Ca^2+^-dependent mechanisms. Given the pleiotropic roles of TRPV4, topical pharmacologic inhibition may therefore represent a promising strategy to reduce MC degranulation, neuropeptide-mediated vasodilation, and pruritus, with the potential to provide benefit beyond current symptomatic treatments.

### 3.3. TRPV4-MC Activation in Decompression Sickness

Decompression sickness is increasingly recognized as a neuro-immune condition in which inflammatory and vascular dysfunction extend beyond the initial formation of inert gas bubbles, with skin lesions representing one of its most visible and clinically informative manifestations [59]. These cutaneous features are characterized by endothelial dysfunction, increased vascular permeability, and robust inflammatory responses following rapid decompression [60]. Although subcutaneous microbubbles are thought to initiate these lesions, the mechanisms by which they translate mechanical stress into sustained inflammation have remained unclear [59,61].

MCs are abundant in the skin and are highly sensitive to mechanical stimuli and well suited to act as early responders to decompression-related stress [62]. Upon activation, MCs release histamine, serotonin, and a broad array of pro-inflammatory mediators that amplify vascular leakage, edema, and tissue inflammation [63,64]. Within this framework, a recent study demonstrated that microbubbles, central to decompression sickness pathology, are sufficient to activate MCs through TRP channel-dependent mechanisms [39]. Exposure of bone marrow–derived MCs to microbubbles induced robust degranulation, marked by increased β-hexosaminidase and histamine release, elevated CD63 expression, and a pronounced rise in intracellular Ca^2+^. Importantly, pharmacologic inhibition of TRPV1 or TRPV4 selectively suppressed both Ca^2+^ influx and MC degranulation, identifying these channels as critical transducers of microbubble-induced mechanical stress [39].

These results provide direct mechanistic evidence that decompression-associated microbubbles engage TRPV4-dependent Ca^2+^ signaling in MCs, linking mechanical forces to MC activation and downstream inflammatory responses in decompression sickness. This illustrates how TRPV4 can function as a mechanosensitive link between physical stimuli and MC-driven neuroimmune inflammation (see Figure 2C). From a therapeutic perspective, microbubble-triggered MC degranulation can be attenuated by blocking TRPV4-dependent Ca^2+^ influx, suggesting that repurposing a clinically tested TRPV4 inhibitor (GSK2798745) as an early systemic adjunct to recompression, delivered orally or intravenously, may suppress TRPV4-mediated inflammatory and vascular leak signaling initiated by bubble–cell mechanotransduction.

## 4. TRPV4 in Other Cell Types and Crosstalk with MCs in Chronic Diseases

While MC-intrinsic TRPV4 signaling contributes to local inflammatory responses, increasing evidence indicates that TRPV4 expressed in other cell types, including epithelial cells, sensory neurons, and endothelial cells, also plays an important role in shaping inflammatory environments. Activation of TRPV4 within these cells can influence the release of inflammatory mediators, ATP, and neuropeptides that subsequently regulate MC recruitment, activation, and mediator release. This bidirectional communication between TRPV4-expressing structural or neuronal cells and MCs promotes neuroimmune crosstalk and can establish feed-forward signaling loops that sustain chronic inflammation. Such intercellular signaling mechanisms are increasingly recognized in systemic disorders in which neuronal and immune pathways intersect, including asthma, irritable bowel syndrome (IBS), and osteoarthritis. In these contexts, TRPV4 activity often operates at the tissue level, where signaling in epithelial, neuronal, or stromal compartments indirectly shapes MC responses and contributes to disease progression. The following sections examine how TRPV4 activity in non–MC populations interact with MCs to influence inflammation and pain across these chronic conditions, while noting that it remains to be determined whether TRPV4 signaling within MCs themselves contributes directly to these processes.

### 4.1. TRPV4 and MC Interactions in Asthma

#### 4.1.1. MCs in Asthma

Asthma is a chronic inflammatory disease marked by airway hyper-responsiveness, bronchoconstriction, and remodeling [65,66,67,68,69]. These pathological features are accompanied by infiltration of inflammatory cells, particularly eosinophils, lymphocytes, and MCs [70]. MCs are widely implicated in both the early-phase allergic response and the long-term remodeling seen in asthma [66]. Upon activation by IgE and antigen, MCs produce mediators like histamine, proteases, LTC4, cytokines, and chemokines that cause airway constriction and edema and recruit other immune cells [66]. During asthma, MC behavior undergoes a marked shift, characterized by increased infiltration into the airway smooth muscle (ASM). Immunohistochemical analyses reveal significantly higher numbers of MCs and lymphocytes within the ASM layer of asthma patients compared to healthy controls, suggesting a role for these immune cells in airway remodeling and hyper-responsiveness [61,63,64,65,66].

#### 4.1.2. TRPV4 in Asthma

**Figure 3:**
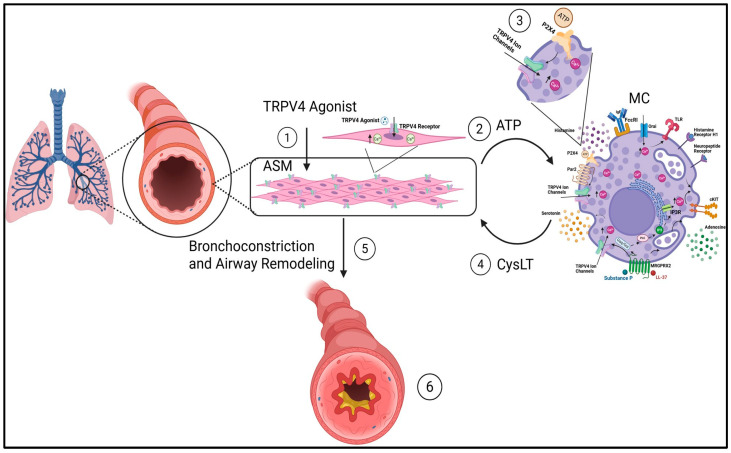
TRPV4–ATP–MC signaling axis in airway smooth muscle contraction and remodeling in asthma. Activation of the mechanosensitive ion channel TRPV4 on airway smooth muscle (ASM) cells ➀ leads to an increase in intracellular Ca^2+^, triggering the release of ATP into the extracellular space ➁. Extracellular ATP acts on purinergic receptors (including P2X4 receptors) expressed on MCs, which are increased in number within ASM bundles in asthmatic airways ➂. P2X activation promotes MC degranulation and the release of cysteinyl leukotrienes (CysLTs) ➃. CysLTs act on ASM to induce contraction of the smooth muscle surrounding the bronchi and bronchioles, resulting in airway narrowing ➄. Sustained activation of this pathway contributes over time to airway remodeling, a hallmark of chronic asthma ➅. Created figure by BioRender (https://app.biorender.com/illustrations/64c169d0c2eb47a439eccfe4?slideId=71ad9fb1-ff62-4b31-bf62-32f07ae9e084, URL accessed on 20 March 2026).

TRPV4 is expressed in various pulmonary cells, including ASM, sensory neurons and MCs [5,12,16,69,71]. TRPV4 plays a role in sensory transduction, contributing to reflexes like cough and regulation of airway tone [16]. ATP, a ubiquitous signaling molecule, plays a bridging role between TRPV4 and downstream neurogenic or inflammatory effects in the airways (Figure 3) [72,73]. It can be released upon TRPV4 activation and subsequently acts on purinergic receptors like P2X3 or P2X4, thereby amplifying inflammation and neural activation in the airway [69,73,74].

Cellular expression of TRPV4 is altered in asthma, and the channel has been implicated in a broad range of airway diseases, including asthma, chronic cough, chronic obstructive pulmonary disease (COPD), pulmonary fibrosis, and pulmonary hypertension [69]. Notably, TRPV4 agonists can depolarize the human vagus nerve, which exhibits high TRPV4 expression, and activate Aδ fibers, leading to cough responses in guinea pigs. These responses are effectively blocked by TRPV4 antagonists, highlighting the channel’s role in sensory nerve activation and its potential as a therapeutic target for airway hyperreactivity and inflammation [69,73]. Although TRPV4 agonists increase intracellular Ca^2+^ in ASM, these increases do not directly cause ASM contraction. Suggesting downstream mediators like ATP may be essential [69]. Activation of TRPV4 by aerosolized agonists like GSK1016790A triggers coughing in conscious guinea pigs, an effect blocked by the TRPV4 antagonist HC067047 and the P2X3 antagonist AF-353, suggesting an MC-adjacent TRPV4–ATP–P2X3 reflex pathway [73,74,75]. TRPV4 activation also induces intracellular Ca^2+^ increases in both nodose neurons and airway smooth muscle cells. However, the resulting ASM contractile effects are MC-dependent in vivo in the guinea pig and human, as well as in guinea pig tracheal tissue. They require the presence of MCs, suggesting that TRPV4-mediated signaling involves a more complex, multicellular cascade that integrates neuronal, smooth muscle, and immune cell interactions [72].

#### 4.1.3. Interaction Between MCs and TRPV4 in Asthma

The most compelling evidence for TRPV4–MC interactions in asthma comes from studies examining their cooperative role in IgE-independent bronchospasm. TRPV4 activation in ASM cells leads to ATP release, which then acts on P2X4 receptors on MCs, causing cysteinyl leukotriene (cysLT) release, and ultimately promoting ASM contraction [72]. In vivo and ex vivo experiments showed that GSK1016790A-induced tracheal contraction required the presence of MCs, as TRPV4 and ATP alone were insufficient. This novel ASM-MC axis, mediated by TRPV4 and ATP, appears to be a new mechanism for non-atopic asthma, where traditional IgE pathways may not be [72]. Collectively, these findings support a model in which TRPV4-dependent signaling contributes to airway inflammation through coordinated interactions between ASM, sensory neurons, and MCs, while emphasizing that MC activation in this context likely arises from integrated multicellular signaling rather than MC-specific TRPV4 activation alone.

#### 4.1.4. Therapeutic Implications

The interaction between TRPV4 and MCs in asthma opens promising therapeutic avenues. TRPV4 antagonists may block not only sensory reflexes like cough but also downstream inflammation and MC-dependent bronchospasm [76,77]. Therapies that inhibit the TRPV4–ATP–P2XR axis could prevent MC-triggered bronchospasm in both atopic and non-atopic asthma [72]. A dual approach, targeting both TRPV4 signaling and MC degranulation, may offer more complete control in severe or refractory asthma phenotypes.

### 4.2. TRPV4-MC Signaling in Visceral Hypersensitivity

#### 4.2.1. MCs Drive Neuro-Immune Crosstalk in IBS

The hallmark of irritable bowel syndrome (IBS) is visceral hypersensitivity (VH), which arises from neuro-immune interactions within the gut. In addition to altered sensory processing, accumulating evidence indicates that low-grade mucosal inflammation, characterized by increased immune cell infiltration and mediator release, contributes to symptom generation in a subset of IBS patients [78]. TRPV4 channels and MCs are central to this process, as MC-derived mediators sensitize sensory neurons, exacerbating pain and gut dysfunction [79,80,81]. Microanatomic studies show that approximately 70% of intestinal MCs are in direct contact with nerves, enabling them to function as both sensory and effector cells. This close neuro-immune interface helps maintain mucosal homeostasis under normal conditions, but in IBS, MCs can disrupt this balance, contributing to visceral hypersensitivity and altered gut function [81].

**Figure 4:**
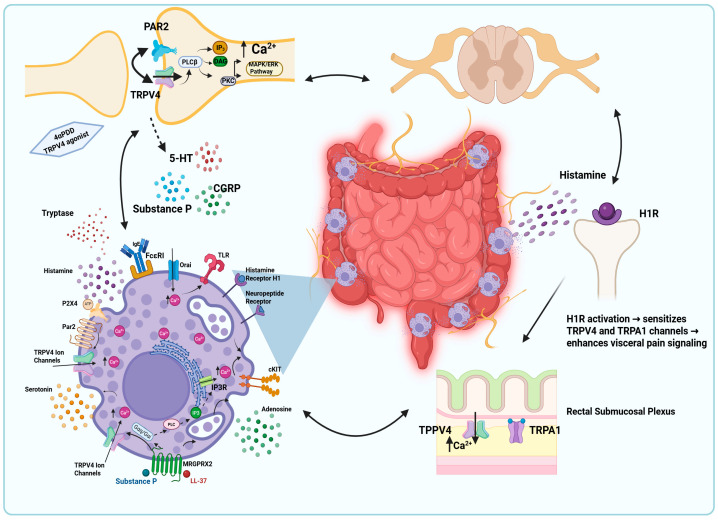
Mast cell–TRP channel neuroimmune crosstalk in visceral hypersensitivity. Histamine and serotonin potentiate TRPV4-dependent Ca^2+^ influx in DRG neurons projecting from the colon by enhancing responses to the TRPV4 agonist 4α-phorbol 12,13-didecanoate (4αPDD). This effect is mediated through activation of PKC, PLCβ, MAPKK, and PLA2 signaling pathways and is associated with increased TRPV4 expression and intracellular Ca^2+^ influx. Enhanced neuronal activation promotes the release of neuropeptides, including CGRP, substance P, and serotonin (5-HT), which further stimulate MC activation and degranulation. MC-derived tryptase activates protease-activated receptor-2 (PAR2) on sensory neurons, leading to TRPV4 sensitization, increased intracellular Ca^2+^ influx, and heightened neuronal excitability. Histamine–H1R–TRP pathway: MC-derived histamine activates histamine H1 receptors (H1R) on sensory nerve endings within the submucosal plexus and dorsal root ganglion (DRG), leading to sensitization of TRPV4 and TRPA1 channels and amplification of visceral pain signaling. (Created figure in BioRender https://app.biorender.com/illustrations/695ec03a4e906d2596f02d29 URL accessed 20 March 2026).

In IBS patients, there is an increase in the number and activation of MCs, with electron microscopy revealing a higher density of degranulated MCs in the colon, often located near enteric nerves. These nerves have been found to contain key mediators, including 5-serotonin (5-HT), calcitonin gene-related peptide (CGRP) and substance P (Figure 4). These mediators are known to induce hypersensitivity in pain-transmitting afferent neurons, contributing to the visceral hypersensitivity observed in IBS [81,82].

Research using mucosal biopsies from IBS patients demonstrated that supernatants from these biopsies, when injected into rats, activated intestinal sensory neurons and induced Ca^2+^ mobilization in dorsal root ganglion neurons. Neuronal activation was inhibited by antihistamines or protease inhibitors, suggesting that some of these MC-derived mediators play a key role in enhancing visceral hypersensitivity in IBS [81,83].

#### 4.2.2. TRPV4 Activation and Mast Cell–Neuron Crosstalk in IBS Pathophysiology

TRPV4 plays a pivotal role in MC–nerve crosstalk in IBS, contributing to visceral hypersensitivity and abdominal pain [84,85]. MCs were frequently localized near mucosal nerve fibers, and only these nerve-adjacent MCs correlated significantly with the severity and frequency of abdominal pain [86]. This neuro–immune interface is highly relevant to TRPV4 signaling, as *TRPV4* mRNA is markedly enriched in colonic sensory neurons and TRPV4 protein is detected in colonic nerve fibers, where it colocalizes with the sensory neuropeptide CGRP. In functional in vivo studies, intracolonic administration of the TRPV4 agonist 4αPDD induced dose-dependent visceral hypersensitivity, assessed by increased abdominal muscle contractions in response to colorectal distension. Conversely, genetic deletion of *Trpv4* or intrathecal delivery of TRPV4-specific siRNA significantly reduced basal visceral nociception as well as hypersensitivity induced by 4αPDD or protease-activated receptor-2 (PAR2) agonists [85,87]. Given that PAR2 is activated by MC-derived proteases such as tryptase, these findings implicate TRPV4 as a downstream effector of MC signaling in sensory neurons. This data supports a mechanistic framework in which MC activation engages PAR2–TRPV4 signaling on colonic sensory afferents, leading to enhanced Ca^2+^ influx, neuronal sensitization, and intestinal hypersensitivity [85]. TRPV4 therefore functions as a convergence point for MC-derived mediators and neuronal signaling pathways in visceral hypersensitivity. Although the extent to which TRPV4 directly regulates MC activity versus acting downstream of MC-neuron communication remains to be determined.

#### 4.2.3. Histamine-Dependent Sensitization of TRPV4 in IBS

Histamine represents a key MC mediator linking immune activation to TRPV4-dependent pain signaling. Histamine released from MCs sensitizes TRPV4 through activation of histamine H1 receptors, enhancing TRPV4-mediated Ca^2+^ influx and neuronal excitability [84]. In IBS patients, both TRPA1 and TRPV4 were shown to be sensitized in the rectal submucosal plexus of IBS patients, an effect replicated in murine dorsal root ganglion neurons using IBS biopsy supernatants and histamine (Figure 4), further confirming the role of H1R [84]. Consistent with these findings, histamine released from MCs in biopsy supernatants from patients with IBS increased intracellular Ca^2+^ concentration in rat primary afferent neurons, supporting a direct role for MC-derived histamine in visceral afferent sensitization [88].

Mechanistic studies further demonstrated the role of histamine and serotonin in potentiating TRPV4 agonist-induced signaling in colonic DRG neurons by activating signaling pathways involving protein kinase C (PKC), phospholipase Cβ (PLCβ), mitogen-activated protein kinase kinase (MAPKK), and phospholipase A2 (PLA2) [79,84,88]. Importantly, intrathecal injection of TRPV4-specific siRNA significantly inhibited the hypersensitivity induced by histamine or serotonin, highlighting the critical role of TRPV4 as a downstream effector of MC-mediator-driven visceral pain [79].

Furthermore, it was determined that TRPV4 is expressed in colonic DRG neurons and is co-expressed with PAR2, a receptor activated by MC tryptase. Activation of PAR2 sensitized TRPV4, leading to increased Ca^2+^ influx and neuronal excitability and visceral hyperalgesia [89]. In contrast, the activation of protease-activated receptor 4 (PAR4) attenuated visceral pain and hypersensitivity in animal models of colorectal distension, suggesting a counter-regulatory pathway that may limit MC-driven nociceptive signaling [90]. PAR4 activation reduced TRPV4-mediated Ca^2+^ signaling in sensory neurons. Notably, MC tryptase activates pronociceptive PAR2, but not PAR4, implying that the balance between these receptors may shape TRPV4-dependent pain outcomes in IBS [90].

Work investigating the brain–gut axis has further highlighted the role of TRPV4 in IBS pathophysiology [91]. In chronic acute combining stress (CACS)-induced IBS model mice, TRPV4 expression was significantly upregulated, suggesting a link between stress and heightened visceral sensitivity [91]. Chemogenetic modulation of the GABAergic pathway from the ACC to the lateral hypothalamic area (LHA) influenced IBS-like symptoms. Activation of this pathway exacerbated symptoms and increased TRPV4 expression and histamine and 5-HT levels, while inhibition of this pathway had the opposite effect. Taken together, these findings highlight TRPV4 as a key integrator of MC-derived inflammatory signals and neuronal sensitization in IBS, while underscoring that TRPV4-dependent effects primarily reflect neuroimmune crosstalk rather than a uniform MC-intrinsic mechanism.

#### 4.2.4. Therapeutic Implications

Accumulating evidence supports functional interactions between MCs and TRPV4 in adjacent cell types that converge on hypersensitivity pathways [92]. Given the well-established elevation and activation of MCs in IBS, further mechanistic studies are critically needed to define how TRPV4 signaling intersects with MC-mediated inflammation and neuroimmune communication and how this interaction contributes to chronic pain and visceral hypersensitivity. Accordingly, targeting TRPV4 or its downstream signaling pathways may provide therapeutic benefit by modulating neuroimmune sensitization, although the widespread expression of TRPV4 necessitates careful consideration of tissue specificity and potential off-target effects.

### 4.3. TRPV4 and MC Crosstalk in Bladder Pain

**Figure 5:**
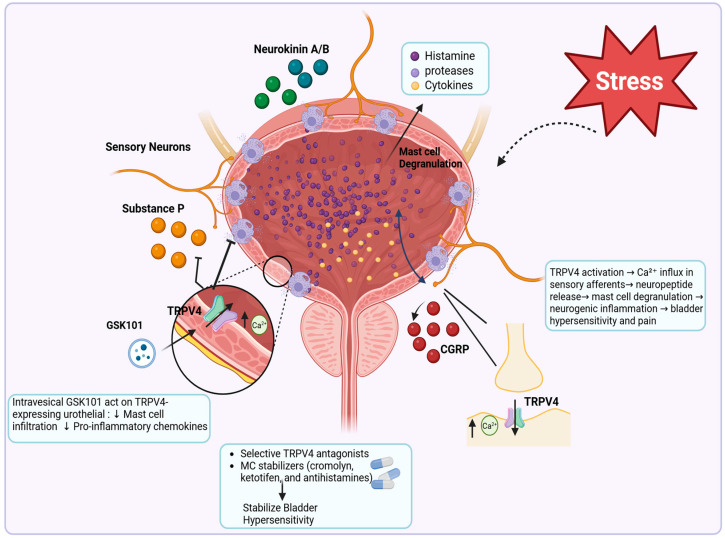
TRPV4–mast cell–neuroimmune signaling in bladder pain. Intravesical activation of TRPV4 on urothelial cells can engage protective mechanisms characterized by reduced MC infiltration and decreased expression of pro-inflammatory chemokines within the bladder wall. Pharmacological modulation using selective TRPV4 antagonists or MC-stabilizing agents (including cromolyn, ketotifen, and antihistamines) further promotes stabilization of MC activity and attenuates bladder hypersensitivity. In contrast, excessive or sustained TRPV4 activation on sensory afferent neurons leads to Ca^2+^ influx and the release of neuropeptides such as substance P (SP), calcitonin gene–related peptide (CGRP), and neurokinins A and B. These neuropeptides trigger MC degranulation, resulting in the release of histamine, proteases, and pro-inflammatory cytokines, thereby driving neurogenic inflammation, bladder hypersensitivity, and pain. (Figure created in BioRender https://app.biorender.com/illustrations/695fc90de72027e3a287818b, URL accessed on 20 March 2026).

Several studies have explored the involvement of TRPV4 channels in bladder hypersensitivity, highlighting their role in modulating bladder inflammation and pain. Intravesical administration of the selective TRPV4 agonist GSK1016790A (GSK101) effectively mitigates LPS-induced bladder inflammation and hypersensitivity in rats [93]. GSK101 treatment reduced MC infiltration and shifted macrophage polarization from the pro-inflammatory M1 phenotype to the anti-inflammatory M2 phenotype, suggesting a regulatory role of TRPV4 in immune responses [93]. Additionally, GSK101 suppressed the upregulation of pro-inflammatory chemokines associated with visceral hypersensitivity (Figure 5). The close spatial and functional association between TRPV4-expressing urothelial and immune cells suggests that TRPV4 signaling may influence MC activity during bladder inflammation. These findings suggest that TRPV4-dependent signaling in bladder pain reflects coordinated interactions between urothelial, neuronal, and immune compartments, in which MC responses may arise secondary to broader tissue-level TRPV4 activation rather than from MC-intrinsic mechanisms alone.

#### Therapeutic Implications

Selective TRPV4 antagonists reduce bladder overactivity and pain in cystitis models [94]. Intravesical administration of the TRPV4 agonist GSK1016790A (GSK101) effectively mitigated LPS-induced bladder inflammation and hypersensitivity in rats, while also reducing MC infiltration, suggesting that controlled TRPV4 activation may engage anti-inflammatory or regulatory pathways under certain conditions. MC-stabilizing agents, including cromolyn, ketotifen, and antihistamines, similarly alleviate inflammation and pain [95]. These findings indicate that both inhibition and context-dependent activation of TRPV4 may provide therapeutic benefit, depending on disease stage, tissue compartment, and downstream signaling pathways engaged. Accordingly, localized or combined therapeutic strategies may maximize efficacy while minimizing systemic exposure. Therefore, targeting both TRPV4 and MCs represents a promising dual approach for effectively managing inflammatory bladder pain syndromes.

### 4.4. Interplay Between TRPV4 and MCs in Osteoarthritis Pathogenesis

**Figure 6:**
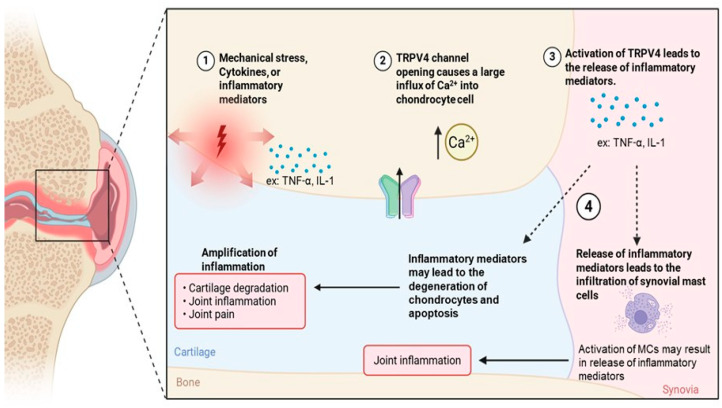
The relationship between osteoarthritis, MCs, and TRPV4. Mechanical stress, cytokines, or inflammatory mediators can promote TRPV4 channel opening. The activation of the TRPV4 channel causes a large influx of Ca^2+^ into chondrocyte cells. Activation of TRPV4 can lead to the release of inflammatory mediators (e.g. TNF-α or IL-1). These inflammatory mediators can influence the infiltration of synovial MCs. When activated, MCs may result in the release of more inflammatory mediators, leading to joint inflammation. In addition to causing the infiltration of MCs, inflammatory mediators may lead to the degeneration of chondrocytes and apoptosis. Chondrocyte degeneration and apoptosis result in the amplification of joint inflammation, pain, and cartilage degradation. Created figure in BioRender (https://app.biorender.com/illustrations/695eb274c5250de2c443a183, URL accessed on 20 March 2026).

Osteoarthritis (OA) is a chronic, progressive joint disease that causes pain, loss of function, and reduced quality of life and is the most common form of arthritis and a leading cause of disability in adults [96]. Once thought to result from simple “wear and tear,” OA is now understood to involve complex inflammatory and metabolic processes [96,97,98]. Inflammation plays a central role in OA progression, where degraded cartilage may trigger immune responses, inducing cytokine production that accelerates further joint damage [99]. MCs, key regulators of inflammation, have emerged as important players in OA and other bone disorders, with recent studies identifying TRPV4 as a diagnostic biomarker for OA that is positively correlated with MC infiltration (Figure 6). This section highlights the interconnected roles of TRPV4 and MCs in OA pathogenesis.

#### Osteoarthritis and MCs and TRPV4

In OA, mechanical stress, cytokines or inflammatory mediators upregulate TRPV4 expression in chondrocyte cells, causing an increase in Ca^2+^ influx [100,101]. Activation of TRPV4 leads to the release of inflammatory mediators such as interleukin-1 and TNF-α, whose downstream signaling contributes to MC infiltration and to OA pathogenesis (Figure 6) [102,103]. MCs participate in the pathogenesis of inflammatory conditions such as osteoarthritis, and increased numbers of MCs are found in patients suffering from these diseases [104]. Clinical studies have shown that both the MC number and the proportion of degranulation MC in the synovium of patients with OA were significantly increased [103,105]. Interestingly, the number of MCs positively correlated with the degree of cartilage injury [105]. The accumulation of inflammatory proteins in MCs may further exacerbate the development and intensity of the inflammatory process associated with OA [90,95,99].

In parallel, inflammatory mediators produced from TRPV4 activation promote chondrocyte degeneration and apoptosis, leading to progressive cartilage damage, joint inflammation, and pain [68]. These findings support a model in which TRPV4-dependent signaling contributes to osteoarthritis progression through multicellular inflammatory pathways involving chondrocytes and immune cells, with MC involvement likely reflecting downstream inflammatory amplification rather than a primary TRPV4-driven MC mechanism. TRPV4 is therefore being explored as a therapeutic target to better understand cartilage biology in OA progression, with emerging evidence demonstrating a positive correlation between TRPV4 expression and MC infiltration [96]. Together, these findings suggest potential new avenues for cartilage regeneration and OA treatment.

### 4.5. MCs and TRPV4 in Neurogenic Inflammation and Migraine Pain

Within trigeminovascular structures, specifically within the meninges, MCs are integral to the initiation and propagation of migraine pain [106,107]. Emerging evidence suggests that MCs may be central to the pathophysiology of migraines, particularly through their interactions with trigeminal afferent and meningeal vasculature. Sami Sbei et al. identified the G protein-coupled receptor MrgprB2 in mice and its human homolog MRGPRX2 as key mediators of migraine-like pain [108]. Utilizing preclinical models, researchers showed that MrgprB2 activation by compounds like PACAP-38 induces migraine-like pain, suggesting a complex interplay between innate immune responses and neuronal signaling.

**Figure 7:**
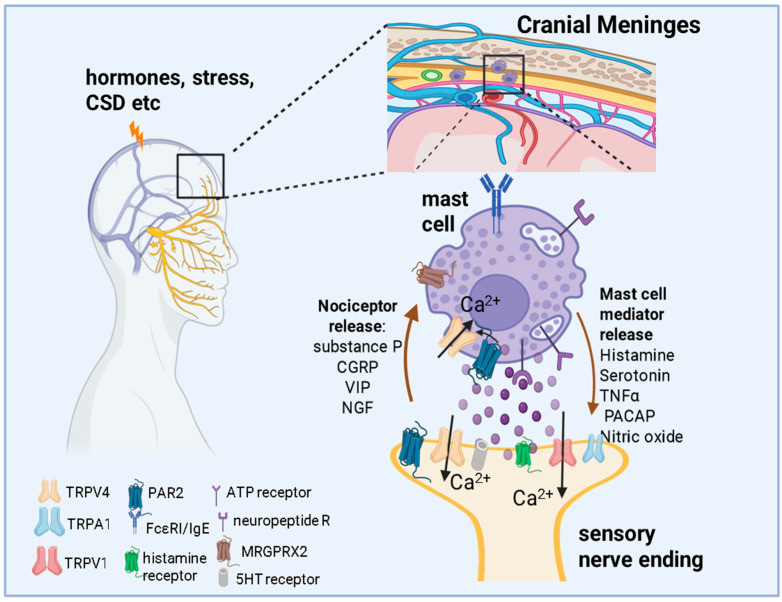
Mast cell–TRPV4 signaling in neurogenic inflammation and migraine pain. Mast cells (MCs) within the meningeal trigeminovascular system are located adjacent to trigeminal sensory afferents and blood vessels, enabling bidirectional neuroimmune communication relevant to migraine. Activation of trigeminal neurons leads to the release of neuropeptides, including CGRP and PACAP, which stimulate MCs via receptors such as MRGPRX2 and CGRP receptors, promoting degranulation and the release of histamine, serotonin, cytokines, proteases, and ATP. These MC-derived mediators sensitize nociceptive afferents and amplify local inflammation. TRPV4 expressed on both MCs and trigeminal sensory neurons mediates Ca^2+^ influx in response to inflammatory, mechanical, and receptor-dependent stimuli, contributing to MC activation and nociceptor sensitization. MC-derived proteases activate PAR2 on sensory neurons, further enhancing TRPV4-dependent signaling and reinforcing feed-forward mechanisms that support peripheral and central sensitization underlying migraine pain. (Figure Created in BioRender https://app.biorender.com/illustrations/62889684e2fd18064a368cd3 URL accessed on 20 March 2026).

In addition to their established role in neuroinflammation and migraine, MCs in the dural meninges interact closely with trigeminal sensory neurons, which, when activated, can release CGRP [36] (Figure 7). MCs themselves express CGRP receptors, positioning them as both targets and modulators of CGRP-driven signaling [109]. CGRP promotes MC degranulation and the release of pro-inflammatory mediators, establishing a feed-forward loop in which MC-derived mediators further sensitize sensory neurons and enhance CGRP signaling [19,110]. In vivo studies provide further support for TRPV4’s involvement in migraine, particularly within the meninges, a tissue rich in MCs. Wei et al. (2011) demonstrated that application of a TRPV4 agonist to the meninges induced facial and hind paw allodynia in rats, a surrogate for migraine-like pain, which was reversed by the TRPV4 antagonist RN1734 [111]. This suggests that TRPV4 activation in the meninges contributes to migraine-relevant nociceptive signaling. Importantly, given the abundance of MCs in the meningeal space, the agonist likely activated TRPV4 not only in nociceptive afferents but also in resident MCs. Such dual activation could amplify local neuroimmune interactions, whereby TRPV4-mediated Ca^2+^ influx in MCs may promote degranulation and the release of pro-inflammatory mediators that further sensitize trigeminal afferents. TRPV4 is also implicated in central sensitization, a process where repeated or sustained stimulation increases the excitability of central pain-processing pathways [112]. This mechanism, along with TRPV4’s trigeminal localization, may explain migraine features such as the characteristic throbbing pain worsened by routine movements, coughing, sneezing, or simple changes in posture [113]. In rat sensory neurons, TRPV4 is co-expressed with CGRP, SP, and PAR2, which is activated by tryptase released from MCs [114].

Zhang and Levy further investigated the role of PAR2 in this context. Activation of PAR2 by the agonist SLIGRL-NH2 sensitized a subset of meningeal nociceptors, an effect enhanced in MC-depleted animals, suggesting that MCs modulate PAR2-mediated neuronal excitability [115]. This sensitization likely involves TRPV channels, including TRPV4, and downstream signaling via protein kinase C pathways. Together, these findings support a neuroimmune framework in which MCs and sensory neurons form a bidirectional signaling circuit that sustains trigeminovascular sensitization. Consistent with this model, pharmacologic blockade of PAR2 with the monoclonal antibody MEDI0618 prevents migraine-like allodynia across multiple preclinical models, including MC-dependent paradigms, identifying PAR2 as a critical upstream regulator of neuroimmune nociceptive signaling [116]. Complementary work shows that direct dural PAR2 activation evokes Ca^2+^ signaling in trigeminal neurons and migraine-like behaviors that are attenuated by PAR2 antagonism or genetic deletion, indicating that MC-derived proteases can drive meningeal nociceptor activation through PAR2-dependent pathways [117]. Ketotifen, an MC-stabilizing histamine H_1_ receptor antagonist, has demonstrated clinical efficacy in migraine prophylaxis, further supporting MC-directed interventions as a viable therapeutic strategy [118]. Combination approaches targeting MC stabilization together with TRPV4 or CGRP signaling may therefore provide additive benefit by suppressing convergent drivers of neurogenic inflammation and trigeminovascular sensitization.

## 5. Summary of Therapeutic Potential and Future Perspectives

Accumulating evidence positions TRPV4 as a regulator of MC-linked neuroimmune signaling across diverse inflammatory and pain-related disorders (Figure 2, Figure 3, Figure 4, Figure 5, Figure 6 and Figure 7; Table 1) [76,77,92,96,111]. Importantly, the extent to which TRPV4 functions in an MC-intrinsic versus multicellular capacity varies by disease context. In LL-37–driven rosacea and microbubble-induced degranulation during decompression sickness, TRPV4 activation directly drives MC Ca^2+^ influx and degranulation, supporting a cell-autonomous model [37,39,55]. In contrast, in asthma, visceral pain, and migraine, TRPV4 activity within epithelial, smooth muscle, or neuronal compartments appears to influence MC responses indirectly through ATP release, purinergic signaling, or mediator amplification loops rather than through MC-restricted mechanisms alone [72,76,79,111].

This distinction has clear therapeutic implications. In settings where MC-intrinsic TRPV4 signaling is strongly supported, localized TRPV4 antagonism may suppress Ca^2+^-dependent exocytosis and downstream inflammatory amplification. In decompression sickness, microbubble-induced mechanical stress activates MC TRPV4, providing a rationale for early TRPV4 inhibition to limit vascular leak and inflammatory signaling [39,119]. In rosacea, topical TRPV4 antagonism may attenuate LL-37–dependent MC activation and neurogenic inflammation while minimizing systemic exposure [37,55].

In asthma and visceral hypersensitivity, TRPV4 appears to operate within a broader multicellular signaling axis. In asthma, TRPV4 activation in airway smooth muscle promotes ATP release and purinergic signaling that contribute to bronchospasm and airway reflex activation [72,76]. In these contexts, targeting the TRPV4-ATP-P2X module may be more appropriate than focusing on MC TRPV4 alone. Preclinical studies demonstrated that aerosolized delivery of TRPV4 antagonists was efficacious and highlighted the potential for localized airway targeting that may reduce systemic exposure [72].

In visceral pain and bladder inflammatory disorders, TRPV4 integrates epithelial, neuronal, and immune signaling [79,84]. A recent study reported that the Chinese herbal formula Shugan Decoction ameliorates IBS-associated colonic dysmotility and visceral hypersensitivity through inhibition of colonic TRPV4–PGE2 signaling [120]. In cystitis models, selective TRPV4 antagonists reduce bladder overactivity, pain, and MC infiltration [94], while MC-stabilizing agents such as cromolyn and ketotifen independently attenuate inflammation and pain [95]. Furthermore, in migraine, MC stabilization has demonstrated clinical relevance [118], underscoring the therapeutic importance of neuroimmune amplification pathways. Within this context, TRPV4-directed strategies may function as complementary therapies. Together, these findings support combination strategies pairing TRPV4 modulation with MC-directed therapies.

Despite encouraging preclinical data, therapeutic translation must account for TRPV4’s pleiotropic physiology. TRPV4 regulates vascular tone, endothelial permeability, osmoregulation, epithelial barrier integrity, and systemic mechanotransduction [94,121,122]. Systemic inhibition therefore carries theoretical risks, including altered vascular reactivity, fluid imbalance, or barrier dysfunction. Although early-phase studies of TRPV4 inhibitors such as GSK2798745 demonstrate acceptable safety and pharmacokinetic profiles [119], and natural compounds such as vitexin and cimifugin have shown TRPV4-inhibitory, antipruritic, and analgesic properties [123,124], feasibility will depend on disease context, duration of exposure, and route of administration. Short-term systemic TRPV4 antagonism may be most appropriate in acute, high-risk settings such as decompression sickness, where rapid suppression of microbubble-induced MC activation and vascular leak could limit secondary injury. Transient systemic inhibition may warrant investigation in severe, refractory pain states where rapid modulation of neuroimmune amplification is hypothesized to be beneficial. In contrast, prolonged systemic blockades in chronic inflammatory or pain disorders may raise safety considerations, highlighting the potential importance of localized delivery or tissue-restricted targeting approaches.

Future progress will depend on (1) precise delineation of MC-intrinsic versus tissue-level TRPV4 mechanisms, (2) targeted or localized delivery strategies that minimize systemic exposure, and (3) rational combination approaches. Overall, TRPV4–MC signaling is best viewed as a context-dependent modulatory network whose pathological relevance varies by cell type, tissue microenvironment, and disease stage. Defining these boundaries will be critical before advancing TRPV4-directed strategies into later-stage clinical development.

## 6. Materials and Methods

This article is a narrative review examining evidence for functional interactions between TRPV4 signaling and MCs in neurogenic inflammation and chronic inflammatory and pain conditions.

### 6.1. Literature Search Strategy

A targeted search of biomedical literature was conducted using PubMed/MEDLINE, Scopus, and Web of Science to identify studies relevant to TRPV4, mast cell activation, and neurogenic inflammation. Search terms were used alone and in combination and included: TRPV4, transient receptor potential vanilloid 4, mast cell, degranulation, MRGPRX2/MRGX2, PAR2, neurogenic inflammation, itch/pruritus, pain, ATP, sensory neuron, trigeminal ganglion, dorsal root ganglion, rosacea, asthma, airway, irritable bowel syndrome/visceral hypersensitivity, bladder pain/interstitial cystitis, osteoarthritis, migraine, acupuncture, and electroacupuncture. Reference lists from included articles, as well as an author-curated reference set, were manually screened to identify additional relevant studies.

### 6.2. Eligibility Criteria

Inclusion criteria: Studies were included if they met all the following conditions: (1) peer-reviewed primary research (in vitro, ex vivo, in vivo animal, or human studies) or scholarly review articles; (2) direct evaluation of TRPV4 (including expression, localization, genetic manipulation, or pharmacologic modulation) and/or assessment of signaling pathways converging on TRPV4 in contexts involving mast cells and/or neuroimmune interactions; (3) reporting of outcomes relevant to MC activation (e.g., Ca^2+^ influx, degranulation, mediator release) and/or neurogenic inflammation (e.g., sensory neuron signaling, itch or pain behaviors, vascular or airway responses, purinergic signaling); and (4) availability of full text in English.

Exclusion criteria: Studies were excluded if they (1) focused on TRP channels other than TRPV4 without TRPV4-specific data; (2) addressed MCs or neurogenic inflammation without a TRPV4-relevant mechanism; (3) were abstracts only, non–peer-reviewed sources or lacked sufficient methodological detail to support mechanistic interpretation; or (4) were not available as full-text articles in English.

### 6.3. Study Selection and Evidence Synthesis

Titles and abstracts were screened for relevance to TRPV4 and MC signaling and neurogenic inflammation. Full texts were reviewed to identify mechanistic links across organ systems, including receptor–channel coupling, Ca^2+^ signaling, MC and other cell mediator release, and ATP/adenosine–purinergic pathways. Evidence was synthesized qualitatively and organized by tissue and cell context and mechanistic pathways.

### 6.4. Data Items Extracted

From each included study, the following data were extracted: model system (species and tissue or cell type); TRPV4 readouts and interventions (agonists, antagonists, knockdown or knockout approaches, and expression changes); MC outcomes (Ca^2+^ responses, degranulation, mediator release); neuroimmune outcomes (itch or pain behaviors, neuronal activation, ATP/adenosine signaling, vascular or airway effects); and pathway-level interpretations relevant to TRPV4–MC interactions.

## Figures and Tables

**Table 1:** Stratified evidence for TRPV4–mast cell signaling across disease contexts.

Condition	Cellular Context	Evidence Type	Experimental Approach	MC- Specific	Key Mechanistic Conclusion	Ref.
Rosacea	MCs (human and mouse)	Genetic + Pharmacologic	TRPV4 siRNA; antagonist HC-067047; LL-37 stimulation; MC degranulation assays	Direct	TRPV4 expression is required for full MC degranulation downstream of MRGPRX2 signaling	[37,55]
Decompression Sickness	MCs (BMMCs) mouse	Pharmacologic	Mechanical microbubble stimulation; TRPV4 antagonists; Ca^2+^ imaging	Direct	Mechanical force induces TRPV4-dependent Ca^2+^ influx, driving MC degranulation	[39]
Acupuncture Analgesia	MCs in skin	Functional (non-genetic)	Needle stimulation at ST36; ATP measurements	Direct (functional)	Mechanical TRPV4 activation in MCs triggers ATP release and purinergic analgesic signaling	[46]
Asthma	Airway smooth muscle; sensory neurons; MCs	Mixed (pharmacologic + inferred MC contribution)	TRPV4 agonists/antagonists; PAR2 activation; guinea pig cough models	Partial/Indirect	TRPV4 drives ATP release and cough reflex via smooth muscle and sensory neurons; MC-derived leukotrienes implicated but MC-autonomous TRPV4 not genetically tested	[69,72,73,75]
Irritable Bowel Syndrome	Sensory neurons; colon; MC-derived mediators	Genetic (neuronal) + inferred MC modulation	*Trpv4* knockout; siRNA; histamine sensitization; biopsy supernatants	Indirect	Neuronal TRPV4 is required for visceral hypersensitivity; MC mediators sensitize TRPV4 but MC-specific TRPV4 deletion not tested	[79,84,91]
Migraine	Meninges; trigeminal ganglion	Pharmacologic (cell type unresolved)	Dural TRPV4 agonist; antagonist RN1734	Unresolved	TRPV4 activation produces headache-like behavior; neuronal vs. MC autonomy not definitively separated	[108,111]
Osteo arthritis	Chondrocytes; joint tissue	Genetic (non-MC) + correlative MC presence	*Trpv4* knockout mice; histology	No direct MC testing	TRPV4 regulates chondrocyte Ca^2+^ signaling and joint degeneration; MC infiltration observed but causal TRPV4–MC axis not established	[96,101]
Bladder Pain	Urothelial cells; immune cells	Pharmacologic (tissue-level)	Intravesical TRPV4 agonist; LPS model	No direct MC testing	TRPV4 modulates inflammatory signaling and hypersensitivity; MC involvement inferred but not directly tested	[93,94]

## Data Availability

No new data were created or analyzed in this study. Data sharing is not applicable to this article.

## Abbreviations

The following abbreviations are used in this manuscript:

TRPV4	Transient Receptor Potential Vanilloid 4
TRP	Transient Receptor Potential
TRPC	Transient Receptor Potential Canonical
TRPM	Transient Receptor Potential Melastatin
TRPA	Transient Receptor Potential Ankyrin
TRPML	Transient Receptor Potential Mucolipin
TRPP	Transient Receptor Potential Polycystin
4α-PDD	4α-phorbol 12,13-didecanoate
MCs	Mast Cells
ARDs	Ankyrin Repeat Domains
ATP	Adenosine Triphosphate
CGRP	Calcitonin Gene-Related Peptide
DRG	Dorsal Root Ganglia
SCF	Stem Cell Factor
CRH	Corticotropin-Releasing Hormone
NT	Neurotensin
FcεRI	Fc epsilon receptor I—High-affinity IgE receptor
IgE	Immunoglobulin E
MrgprB2	Mas-related G Protein–Coupled Receptor B2 (mouse)
MRGPRX2	Mas-related G Protein–Coupled Receptor X2 (human)
LL-37	Cathelicidin Antimicrobial Peptide
HC067047	Selective TRPV4 Antagonist (compound)
VIP	Vasoactive Intestinal Peptide
PACAP	Pituitary Adenylate Cyclase-Activating Polypeptide
PACAP1-38	Pituitary Adenylate Cyclase-Activating Polypeptide 1-38
AD	Atopic Dermatitis
PN	Prurigo Nodularis
SP	Substance P
NGF	Nerve Growth Factor
BDNF	Brain-Derived Neurotrophic Factor
ASM	Airway Smooth Muscle
LTC4	Leukotriene C4
OVA	Ovalbumin
TMDCD	Tuo-Min-Ding-Chuan Decoction
P2X3	Purinergic Receptor P2X, Ligand-Gated Ion Channel 3
P2X4	Purinergic Receptor P2X, Ligand-Gated Ion Channel 4
COPD	Chronic Obstructive Pulmonary Disease
Aδ fibers	A-delta fibers (a type of sensory nerve fiber)
GSK1016790	A Selective TRPV4 Agonist (compound)
AF-353	Selective P2X3 Receptor Antagonist (compound)
[Ca^2+^]i	Intracellular Calcium Concentration
VH	Visceral Hypersensitivity
GI	Gastrointestinal
5-HT	5-Hydroxytryptamine (Serotonin)
TNBS	2,4,6-Trinitrobenzene Sulfonic Acid
MPO	Myeloperoxidase
RMCP-2	Rat MC Protease-2
PKC	Protein Kinase C
PLCβ	Phospholipase C Beta
MAPKK	Mitogen-Activated Protein Kinase Kinase
PLA2	Phospholipase A2
siRNA	Small Interfering RNA
PAR2	Protease-Activated Receptor 2
PAR4	Protease-Activated Receptor 4
ACC	Anterior Cingulate Cortex
GABAergic	Gamma-Aminobutyric Acid-Producing (Neurons)
LPS	Lipopolysaccharide
OA	Osteoarthritis
EA	Electroacupuncture
